# Inflammatory–Molecular Clusters as Predictors of Immunotherapy Response in Advanced Non-Small-Cell Lung Cancer

**DOI:** 10.3390/jcm15010349

**Published:** 2026-01-02

**Authors:** Vlad Vornicu, Alina-Gabriela Negru, Razvan Constantin Vonica, Andrei Alexandru Cosma, Mihaela Maria Pasca-Fenesan, Anca Maria Cimpean

**Affiliations:** 1 Doctoral School in Medicine, “Victor Babes” University of Medicine and Pharmacy, 300041 Timisoara, Romania; vlad.vornicu@umft.ro (V.V.); mihaela.fenesan@umft.ro (M.M.P.-F.); 2 Department of Oncology, ONCOHELP Hospital Timisoara, Ciprian Porumbescu Street, No. 59, 300239 Timisoara, Romania; 3 Department of Cardiology, “Victor Babes” University of Medicine and Pharmacy, 300041 Timisoara, Romania; alinanegru@umft.ro; 4 Preclinical Department, Faculty of Medicine, “Lucian Blaga” University of Sibiu, 550169 Sibiu, Romania; razvanconstantin.vonica@ulbsibiu.ro; 5 Department of Microscopic Morphology/Histology, “Victor Babes” University of Medicine and Pharmacy, 300041 Timisoara, Romania; acimpeanu@umft.ro; 6 Center of Expertise for Rare Vascular Disease in Children, Emergency Hospital for Children Louis Turcanu, 300011 Timisoara, Romania; 7 Center of Genomic Medicine, “Victor Babes” University of Medicine and Pharmacy, 300041 Timisoara, Romania; 8 Research Center for Pharmaco-Toxicological Evaluation, “Victor Babes” University of Medicine and Pharmacy, 300041 Timisoara, Romania

**Keywords:** non-small-cell lung cancer (NSCLC), immune checkpoint inhibitors (ICIs), systemic inflammatory indices, neutrophil-to-lymphocyte ratio (NLR), PD-L1 expression, EGFR/KRAS mutations, prognostic biomarkers, immunotherapy outcomes

## Abstract

**Background/Objectives**: Immunotherapy has improved outcomes for selected patients with advanced non-small-cell lung cancer (NSCLC), yet the predictive value of individual biomarkers such as PD-L1 remains limited. Systemic inflammatory indices derived from routine blood tests may complement molecular and immunohistochemical features, offering a broader view of host–tumor immunobiology. **Methods**: We conducted a retrospective study of 298 patients with stage IIIB–IV NSCLC treated with immune checkpoint inhibitors (ICIs) at a tertiary oncology center between 2022 and 2024. Baseline neutrophil-to-lymphocyte ratio (NLR), platelet-to-lymphocyte ratio (PLR), lymphocyte-to-monocyte ratio (LMR), and systemic immune–inflammation index (SII) were collected alongside PD-L1 expression and molecular alterations (EGFR, KRAS, ALK, TP53). Patients were stratified into inflammatory–molecular clusters integrating these parameters. Associations with objective response rate (ORR), progression-free survival (PFS), and overall survival (OS) were evaluated using Kaplan–Meier and multivariate Cox analyses. **Results**: Four distinct inflammatory–molecular clusters demonstrated significantly different outcomes (*p* < 0.001). Patients with low NLR and high PD-L1 expression (Cluster A) showed the highest ORR (41%), longest median PFS (13.0 months), and OS (22.5 months). The EGFR/ALK-driven, inflammation-dominant cluster (Cluster C) exhibited poor response (ORR 7%) and shortest survival (PFS 4.3 months). High NLR (HR 2.12), PD-L1 < 1% (HR 1.91), and EGFR mutation (HR 2.36) independently predicted shorter PFS. A combined model incorporating NLR, PD-L1, and molecular status outperformed individual biomarkers (AUC 0.82). **Conclusions**: Integrating systemic inflammatory indices with PD-L1 expression and molecular alterations identifies clinically meaningful NSCLC subgroups with distinct immunotherapy outcomes. This multidimensional approach improves prediction of ICI response and may enhance real-world patient stratification, particularly in settings with limited access to extended molecular profiling.

## 1. Introduction

In recent years, immunotherapy has redefined the therapeutic paradigm of advanced non-small-cell lung cancer (NSCLC), offering durable clinical responses and prolonged survival in selected patients [1]. Despite these remarkable advances, only a fraction of individuals experiences sustained benefit, while many exhibit primary or early acquired resistance. This marked variability highlights the urgent need for reliable biomarkers capable of anticipating treatment outcomes and guiding patient selection with greater precision [2].

Lung cancer remains one of the leading causes of cancer-related mortality worldwide and represents a biologically heterogeneous group of malignancies with distinct histological, molecular, and clinical characteristics. The two major histologic categories are NSCLC, accounting for approximately 85% of cases, and small cell lung cancer (SCLC), a neuroendocrine subtype characterized by rapid growth, early dissemination, and a unique therapeutic paradigm [3]. While immunotherapy has become a cornerstone in the management of advanced NSCLC, particularly in tumors expressing PD-L1 or lacking actionable driver mutations, its role in SCLC differs substantially and is influenced by distinct tumor biology and immune microenvironment features. SCLC is typically associated with high tumor proliferation rates, widespread genomic instability, pronounced neuroendocrine differentiation, and specific patterns of systemic inflammation that are not directly comparable to those observed in NSCLC. Moreover, treatment strategies, response dynamics, and prognostic biomarkers differ fundamentally between the two entities. To ensure biological and clinical homogeneity and to allow a meaningful interpretation of inflammatory–molecular interactions in the context of immunotherapy, the present study deliberately focused on patients with advanced NSCLC, excluding SCLC cases from the analysis [4].

Among the currently established predictive markers, programmed death-ligand 1 (PD-L1) expression remains the most widely implemented in clinical practice [5]. However, its predictive accuracy is limited. A substantial proportion of patients with high PD-L1 expression fail to respond to immune checkpoint inhibitors (ICIs), whereas others with low or absent expression achieve unexpected and durable remission [6]. This variation indicates that PD-L1 alone does not adequately capture the broader interactions between the tumor and the host immune system [7]. The efficacy of immunotherapy likely depends on a broader spectrum of factors, including systemic inflammation, immune cell composition, and tumor-intrinsic molecular alterations [8].

Systemic inflammatory indices derived from routine complete blood count (CBC) testing, including the neutrophil-to-lymphocyte ratio (NLR), platelet-to-lymphocyte ratio (PLR), lymphocyte-to-monocyte ratio (LMR), and the systemic immune–inflammation index (SII), have emerged as accessible and cost-effective markers of immune–inflammatory equilibrium [9]. Systemic inflammation reflects a state of immune dysregulation resulting from sustained tumor–host interactions and plays a central role in cancer progression. In advanced non-small-cell lung cancer (NSCLC), these indices provide surrogate markers of the balance between pro-tumor inflammatory activity and antitumor immune competence. Elevated neutrophil- and platelet-based indices, such as NLR, PLR, and SII, are generally associated with a pro-tumorigenic inflammatory state characterized by cytokine release, angiogenesis, and suppression of cytotoxic lymphocyte function, whereas higher lymphocyte-related indices, including LMR, reflect improved immune surveillance and more favorable outcomes. Although these markers offer valuable insight into systemic immune status, their performance when evaluated in conjunction with molecular alterations and immunohistochemical features, such as PD-L1 expression, remains incompletely characterized [10]. These systemic inflammatory changes also interact with tumor-intrinsic molecular drivers, including EGFR, KRAS, ALK, and TP53 mutations, influencing immune evasion, disease progression, and response to immune checkpoint inhibitors [11].

Interactions between inflammatory cell populations and tumor molecular drivers, such as EGFR, KRAS, ALK, or TP53 mutations, can shape the tumor microenvironment, alter cytokine signaling, and modulate the degree of immune activation or suppression [12]. For instance, KRAS-driven tumors have been associated with neutrophil-rich inflammation and distinct metastatic behavior, while EGFR-mutated neoplasms frequently display “cold” immune phenotypes, characterized by low PD-L1 expression and reduced sensitivity to checkpoint blockade [13]. These associations suggest that the integration of inflammatory and molecular features may provide a more comprehensive understanding of therapeutic responsiveness in NSCLC [14]. Recent evidence supports the concept that composite biomarkers, combining systemic inflammatory markers, PD-L1 expression, and molecular alterations, outperform single predictors in estimating both survival and response to ICIs [15]. Nevertheless, data from real-world, homogeneous cohorts remain limited, especially in Central and Eastern European populations, where access to comprehensive molecular profiling may vary [16].

The present study investigates the interplay between systemic inflammatory indices and molecular characteristics in patients with advanced NSCLC treated with immune checkpoint inhibitors. By defining distinct inflammatory–molecular clusters and evaluating their association with therapeutic response, progression-free survival, and overall survival, this analysis seeks to delineate clinically relevant profiles that may enhance the personalization of immunotherapy strategies. A clearer understanding of how systemic inflammation, molecular alterations, and PD-L1 expression interact may help refine patient selection for immunotherapy. In this study, we evaluated these factors simultaneously to identify clinically relevant subgroups within a real-world NSCLC population treated at a tertiary center. Our aim was to develop a practical framework that reflects routine clinical data rather than theoretical models.

## 2. Materials and Methods

### 2.1. Study Design and Setting

This retrospective, observational study was conducted at the OncoHelp Medical Center in Timișoara, Romania, a specialized oncology facility providing comprehensive diagnostic and therapeutic services for solid malignancies. The analysis focused on patients with advanced NSCLC treated with ICIs between June 2022 and December 2024. The primary objective was to investigate the association between systemic inflammatory markers, molecular alterations, and immunotherapy outcomes.

All data were retrieved from the institutional electronic medical record system using a standardized protocol designed to ensure accuracy and consistency. Ethical approval was obtained from the OncoHelp Medical Center Ethics Committee (approval number 1186/07.05.2025). The requirement for informed consent was waived due to the retrospective nature of the study and the anonymization of all patient records.

### 2.2. Patient Selection

Eligibility criteria were defined according to international guidelines and previous studies (Table 1).

### 2.3. Data Collection

For all eligible patients, demographic, clinical, laboratory, and molecular data were extracted and curated in a de-identified dataset.

Demographic variables included age, sex, place of residence (urban/rural), smoking history, and ECOG performance status. Clinical parameters encompassed tumor histology, anatomical location of the primary lesion (central or peripheral), and disease stage at the start of immunotherapy.

Molecular testing was performed according to institutional protocols and included analysis for EGFR mutations, KRAS mutations, ALK rearrangements, and TP53 alterations, using PCR-based or next-generation sequencing methods. PD-L1 expression was determined by immunohistochemistry and reported as tumor proportion score (TPS), classified into <1%, 1–49%, and ≥50% categories.

Laboratory data were obtained from baseline CBC results collected within seven days prior to the first immunotherapy dose. The following parameters were recorded: absolute neutrophil, lymphocyte, monocyte, eosinophil, and platelet counts. From these, systemic inflammatory indices were calculated:

- Neutrophil-to-Lymphocyte Ratio (NLR) = neutrophils/lymphocytes;
- Platelet-to-Lymphocyte Ratio (PLR) = platelets/lymphocytes;
- Lymphocyte-to-Monocyte Ratio (LMR) = lymphocytes/monocytes;
- Systemic Immune–Inflammation Index (SII) = (neutrophils × platelets)/lymphocytes.

Baseline blood samples were obtained from peripheral venous blood collected under routine clinical conditions within seven days prior to the initiation of immunotherapy. CBC analyses were performed on ethylenediaminetetraacetic acid (EDTA)-anticoagulated samples using automated hematology analyzers in the institutional laboratory of the OncoHelp Medical Center, in accordance with internal quality-controlled protocols and national laboratory standards. Absolute neutrophil, lymphocyte, monocyte, eosinophil, and platelet counts were recorded, and systemic inflammatory indices were calculated using standardized formulas: NLR, PLR, LMR, and systemic immune–inflammation index (SII).

Tumor tissue samples were obtained from diagnostic biopsy or surgical specimens and processed according to institutional histopathology protocols. Formalin-fixed, paraffin-embedded (FFPE) tissue sections were used for immunohistochemical and molecular analyses. PD-L1 expression was assessed by immunohistochemistry using clinically validated assays approved for routine diagnostic use at the institution, and results were reported as TPS, categorized as <1%, 1–49%, or ≥50%, in line with international guidelines. Molecular testing for EGFR mutations, KRAS mutations, ALK rearrangements, and TP53 alterations was performed using polymerase chain reaction (PCR)-based methods or next-generation sequencing platforms, depending on tissue availability and testing period, following standardized institutional protocols. All laboratory and pathology analyses were conducted as part of routine clinical care by certified personnel, ensuring reproducibility and consistency across the study cohort.

### 2.4. Treatment and Follow-Up

Patients received immunotherapy either as first-line monotherapy in PD-L1–high tumors (TPS ≥ 50%) or as combination therapy with platinum-based doublets in cases of lower PD-L1 expression or rapid disease progression. Standard dosing regimens were used according to national and ESMO guidelines in effect during the study period.

Radiological evaluation of treatment response was performed every 9–12 weeks using computed tomography (CT) or PET-CT scans, and responses were classified according to the Response Evaluation Criteria in Solid Tumors (RECIST 1.1) as complete response (CR), partial response (PR), stable disease (SD), or progressive disease (PD).

#### Immunotherapy Regimens

Among the 298 patients included in the analysis, immune checkpoint inhibitors were administered according to standard clinical practice. Pembrolizumab was the most frequently used agent, followed by nivolumab, atezolizumab, and durvalumab. Pembrolizumab was administered at a dose of 200 mg every 3 weeks or 400 mg every 6 weeks, nivolumab at 240 mg every 2 weeks or 480 mg every 4 weeks, atezolizumab at 1200 mg every 3 weeks, and durvalumab at 1500 mg every 4 weeks.

Immunotherapy was used as monotherapy predominantly in patients with high PD-L1 expression (tumor proportion score ≥ 50%), while combination regimens with platinum-based chemotherapy were more frequently employed in patients with lower PD-L1 expression or aggressive disease at presentation. All dosing schedules reflected approved regimens and institutional protocols, and no dose reductions of immune checkpoint inhibitors were required during treatment.

### 2.5. Outcomes

The primary endpoint was progression-free survival (PFS), defined as the time from the initiation of immunotherapy to radiologically confirmed progression or death from any cause. The secondary endpoint was overall survival (OS), measured from treatment initiation to death or last known follow-up. Objective response rate (ORR = CR + PR) was also evaluated.

Patients were stratified into inflammatory–molecular clusters based on combinations of systemic indices (NLR, PLR, LMR, SII) and molecular/immunohistochemical features (PD-L1 expression, EGFR/KRAS/ALK/TP53 status). Cluster behavior was correlated with survival outcomes and treatment response.

### 2.6. Statistical Analysis

All statistical analyses were performed using IBM SPSS Statistics version 26 and GraphPad Prism version 9.0. Continuous variables were expressed as mean ± standard deviation (SD) or median (interquartile range, IQR) and compared using independent-samples t-tests or Mann–Whitney U tests, depending on distribution. Categorical data were analyzed using the χ^2^ test or Fisher’s exact test.

Optimal cut-off values for inflammatory indices were determined using receiver operating characteristic (ROC) curve analysis based on progression status at 6 months. Survival curves were estimated by the Kaplan–Meier method and compared using the log-rank test. Independent prognostic factors for PFS and OS were identified through Cox proportional-hazards regression modeling. Variables with *p* < 0.10 in univariate analysis were included in multivariate models.

All statistical tests were two-tailed, and *p* < 0.05 was considered statistically significant. Results were reported in accordance with the STROBE guidelines for observational studies.

## 3. Results

### 3.1. Baseline Characteristics

The study cohort included 298 patients with advanced NSCLC who received ICIs between 2022 and 2024. The majority were men in their mid-sixties, predominantly former or current smokers, with preserved performance status at baseline. Adenocarcinoma was the most common histologic subtype, followed by squamous cell carcinoma and large-cell carcinoma. Molecular profiling revealed a heterogeneous genomic background, including a small proportion of EGFR-, KRAS-, and ALK-positive tumors, alongside a larger subgroup harboring TP53 alterations. Approximately one third of patients exhibited high PD-L1 expression (≥50%), while another third showed intermediate expression (1–49%), reflecting a balanced distribution typical of real-world immunotherapy populations. A complete summary of baseline characteristics is provided in Table 2.

### 3.2. Baseline Inflammatory Indices

At baseline, systemic inflammatory indices exhibited significant variability across the study cohort and showed a clear association with PD-L1 expression levels. Patients with low PD-L1 expression (<1%) demonstrated significantly higher neutrophil-to-lymphocyte ratio (NLR) values compared with those with intermediate (1–49%) and high (≥50%) PD-L1 expression (median NLR: 6.0 vs. 5.0 vs. 3.8, respectively; *p* = 0.018). Similarly, platelet-to-lymphocyte ratio (PLR) values were progressively lower with increasing PD-L1 expression (median PLR: 255 vs. 243 vs. 218; *p* = 0.032).

In contrast, lymphocyte-to-monocyte ratio (LMR) showed an inverse pattern, with significantly higher values observed in patients with high PD-L1 expression compared with those with lower PD-L1 levels (median LMR: 2.8 vs. 2.3 vs. 2.0; *p* = 0.011). The systemic immune–inflammation index (SII) followed a similar trend, with the highest values recorded in the PD-L1–negative group and the lowest in patients with PD-L1 ≥ 50% (median SII: 1180 vs. 1020 vs. 880; *p* = 0.024) (Table 3).

These findings indicate that lower PD-L1 expression is associated with a higher systemic inflammatory burden at treatment initiation, whereas tumors with higher PD-L1 levels tend to be accompanied by more favorable inflammatory profiles, characterized by lower neutrophil- and platelet-based indices and higher lymphocyte-related parameters.

### 3.3. Inflammatory–Molecular Clusters

By integrating systemic inflammation with PD-L1 status and driver mutations, four distinct inflammatory–molecular clusters were defined. The first cluster combined low NLR, high PD-L1 expression, and a wild-type molecular background, representing an immune-active phenotype. The second and third clusters, characterized by elevated NLR and the presence of KRAS, TP53, or EGFR/ALK alterations, reflected more immunosuppressed or genomically driven profiles. The fourth cluster displayed intermediate values without a dominant molecular signal, bridging the inflammatory and non-inflammatory groups. An overview of cluster composition is presented in Table 4.

### 3.4. Response to Immunotherapy

Clinical response differed substantially across the four clusters. Patients with low baseline inflammation and high PD-L1 expression demonstrated the most favorable outcomes, with higher objective and disease control rates. In contrast, those combining a strong systemic inflammatory profile with low PD-L1 levels and EGFR or ALK mutations responded poorly. The intermediate clusters exhibited mixed outcomes, aligning with their heterogeneous biological characteristics. Objective response rates differed significantly across clusters, ranging from 41.0% in Cluster A to 7.0% in Cluster C (*p* < 0.001), in line with the observed survival differences. The same pattern was observed when evaluating both objective responses and overall disease control, as summarized in Table 5.

### 3.5. Survival Outcomes

**Figure 1:**
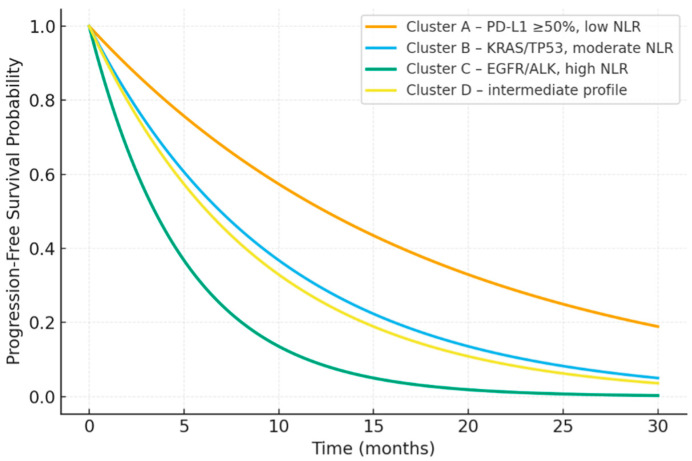
Progression-free survival across inflammatory–molecular clusters.

After a median follow-up exceeding one-year, significant survival differences were observed among the inflammatory–molecular clusters (Figure 1). Kaplan–Meier analysis demonstrated clear separation of progression-free survival curves across the four inflammatory–molecular clusters (Figure 1). Median Progression-free survival (PFS) was longest in Cluster A (13.0 months), followed by Cluster D (9.1 months) and Cluster B (8.4 months), while Cluster C exhibited the shortest median PFS (4.3 months). The difference among progression-free survival curves was statistically significant (log-rank test, *p* < 0.001). The partially overlapping progression-free survival curves observed for Clusters B and D likely reflect shared intermediate-risk characteristics. Both clusters exhibit moderate programmed death-ligand 1 (PD-L1) expression and inflammatory profiles that are neither strongly immune-active nor profoundly immune-suppressed, resulting in comparable clinical outcomes under immune checkpoint inhibition.

The immune-active subgroup characterized by low neutrophil-to-lymphocyte ratio (NLR) and high PD-L1 expression also achieved the longest overall survival (median overall survival (OS): 22.5 months), whereas the EGFR/ALK-driven, inflammation-dominant cluster showed the poorest outcomes (median OS: 9.2 months). Overall survival differences among clusters were statistically significant (log-rank test, *p* = 0.003). The full multivariate model is presented in Table 6.

### 3.6. Predictive Model Performance

**Figure 2:**
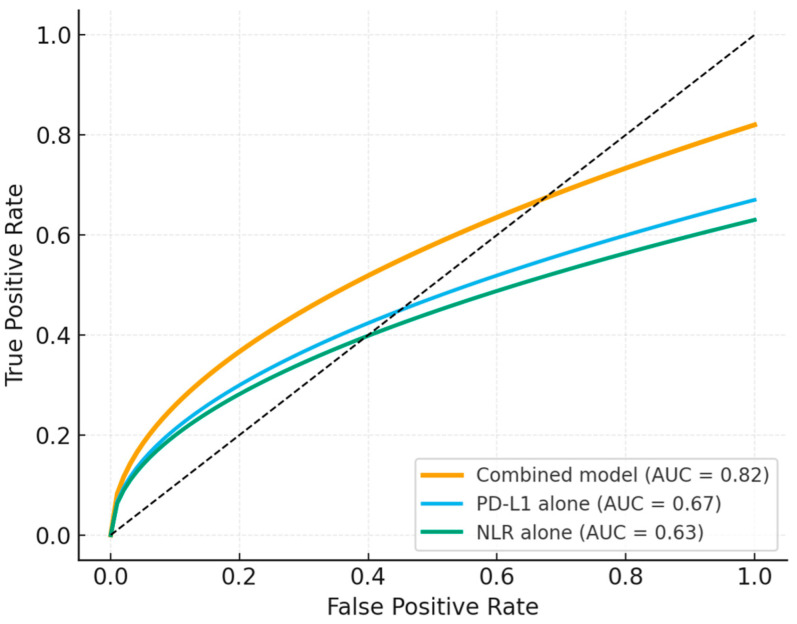
Predictive performance of the combined inflammatory–molecular model compared with PD-L1 and NLR alone.

When combined into an integrated model, inflammatory and molecular parameters provided stronger predictive power for immunotherapy response than any single variable alone. The composite index incorporating NLR, PD-L1, and mutation status showed the highest discriminative accuracy on ROC analysis, outperforming individual markers. This suggests that a multidimensional approach, reflecting both systemic immune balance and tumor-intrinsic biology, offers a more reliable framework for treatment prediction (Figure 2).

## 4. Discussion

In our cohort, the combination of systemic inflammatory markers and tumor molecular features showed a clear impact on immunotherapy outcomes [20]. Patients with low NLR and high PD-L1 expression experienced the most favorable responses and survival, while those with pronounced systemic inflammation or specific oncogenic drivers, particularly EGFR mutations, had substantially poorer results [21]. These data support the idea that routinely available clinical parameters, when interpreted together, can provide meaningful predictive information.

From a clinical standpoint, the observation that patients with low NLR and high PD-L1 expression derived the greatest benefit from ICIs is consistent with prior evidence that systemic inflammation shapes immune efficacy. Elevated NLR has long been recognized as a marker of immune suppression and tumor-promoting inflammation. Studies demonstrated that high baseline NLR correlates with poor outcomes under PD-1/PD-L1 blockade, independently of tumor burden or performance status [22]. The present results corroborate previous findings, indicating that an inflamed systemic milieu, rich in neutrophils and thrombocytes but poor in lymphocytic activity, can attenuate immunotherapeutic benefit [23]. In contrast, patients with lower NLR and higher LMR appear to maintain a more effective antitumor immune response.

At the molecular level, the results from our cohort echoed patterns described in previous studies, but with some nuances. Patients with EGFR-mutated tumors generally had low PD-L1 expression and higher inflammatory indices at baseline, which corresponded to limited benefit from immune checkpoint inhibitors [24]. KRAS-mutated tumors showed more variable inflammatory profiles, and outcomes were similarly heterogeneous, consistent with the biological diversity of this subtype [25]. Although TP53 alterations were frequent, their influence on treatment response in our dataset appeared modest when compared with the effects of NLR or PD-L1 status [26,27]. These differences suggest that inflammatory markers and molecular data should be interpreted together, not as separate components.

By comparison, KRAS-mutated tumors demonstrated an intermediate pattern, with moderate PD-L1 expression and variable inflammatory indices [28]. This heterogeneity reflects the molecular diversity of KRAS-driven NSCLC [29]. Some KRAS-mutant tumors, particularly those co-mutated with TP53, tend to generate a highly immunogenic microenvironment and respond favorably to PD-1 inhibitors [30], while others with STK11 or KEAP1 co-mutations exhibit profound immunoresistance [31]. Although co-mutation profiling was not available in this study, the correlation between KRAS and elevated inflammatory markers (notably NLR and PLR) suggests a link between RAS-mediated cytokine signaling and systemic inflammation [32].

Certain histopathologic patterns also appeared to align with the inflammatory profiles observed in our study. Adenocarcinomas, which were predominant in our cohort, more often fell into clusters with lower systemic inflammation and higher PD-L1 expression, corresponding to better clinical outcomes [33,34]. In contrast, tumors with more extensive necrosis or central location tended to present with higher NLR and SII values [35]. Although not conclusive, the patterns we observed imply that certain morphologic characteristics might relate to the patient’s baseline immune state [36].

The morphopathologic interplay between the tumor microenvironment and systemic immunity provides an important explanatory layer for these data. Chronic exposure to inflammatory cytokines (IL-1β, IL-6, TNF-α) induces epithelial–mesenchymal transition (EMT), promotes angiogenesis, and fosters resistance to immune clearance [37]. Morphologically, such tumors often display irregular glandular differentiation, desmoplastic stroma, and infiltration by suppressive immune cells such as tumor-associated macrophages (TAMs) and neutrophils [38]. In the present cohort, patients with high SII and PLR values (reflecting enhanced platelet and neutrophil activity), likely harbored tumors with similar stromal reprogramming. These morphologic features have been linked in previous work to reduced responsiveness to immunotherapy [2].

Clinically, these pathobiological observations translate into distinct trajectories under immunotherapy. The immune-active cluster (low NLR, high PD-L1, wild type genotype) experienced the most durable disease control, reinforcing the concept that a balanced host–tumor immune axis is essential for sustained response [39]. The immune-repressed, EGFR/ALK-driven cluster progressed rapidly, highlighting the limitations of checkpoint blockade in genomically defined subtypes with poor immune visibility [25]. Interestingly, the intermediate clusters, particularly those with KRAS or TP53 mutations, showed partial benefit, indicating that inflammatory markers could help differentiate prognosis even among patients with similar molecular profiles [29,40].

Looking at PD-L1 together with systemic inflammation shows how local tumor factors and the overall immune state interact in shaping treatment response [41]. PD-L1 quantifies the tumor’s adaptive immune evasion mechanism, whereas NLR and related indices reflect the host’s baseline inflammatory tone [42]. Combining these dimensions allows a more complete estimation of immune equilibrium. Similar composite models have been proposed in other studies, which emphasized that systemic markers capture longitudinal immune dynamics that static tumor biopsies may miss [43,44]. The present analysis confirms that such an integrative approach increases predictive accuracy (AUC = 0.82), outperforming PD-L1 or NLR alone.

The associations between high systemic inflammation and poor immunotherapy response in our cohort are consistent with established immunologic mechanisms. Elevated neutrophil counts and reduced lymphocyte levels may indicate an imbalance that limits effective antitumor immunity. Similarly, increased platelet activity, reflected in higher PLR and SII value, has been linked to pro-tumor signaling pathways. Although our study was not designed to explore these mechanisms directly, the clinical patterns observed support the relevance of these pathways in routine practice [45,46,47,48,49].

Because this analysis reflects routine practice, the findings may be directly relevant for everyday clinical decision-making: routine hematologic and immunohistochemical markers, already available in standard care, can be strategically combined to refine patient selection for immunotherapy [50]. This is particularly relevant in regions where next-generation sequencing or tumor mutational burden testing remains limited [51]. In such environments, simple composite markers may offer a pragmatic surrogate for complex genomic assays, guiding therapeutic decisions without additional cost or delay [52].

When compared with prior studies, our results reinforce and broaden existing knowledge on these relationships. First, histopathologic and molecular characteristics dictate the intrinsic immune phenotype of NSCLC, as shown by prior studies, which correlated genomic load and immune infiltration with checkpoint sensitivity [53,54]. Second, systemic inflammation modifies the functional state of this immune phenotype, either amplifying or suppressing its expression [50]. The present work bridges these two dimensions, showing that inflammation and molecular status are not parallel phenomena but converging forces shaping therapeutic outcomes [52].

Pathologically, the clusters we identified may represent a spectrum of biological behaviors rather than sharply separated groups [54]. Cluster A resembles the “inflamed immune-rich” subtype, marked by high PD-L1, active cytotoxic infiltration, and low systemic inflammation [55,56]. Cluster C parallels the “immune desert” profile, with genomic addiction, sparse lymphocyte infiltration, and high neutrophil predominance [57,58]. The intermediate clusters mirror transitional phenotypes, where local immune engagement exists but is counterbalanced by systemic pro-inflammatory signals [59]. These observations suggest that, in selected patients, combining ICIs with anti-inflammatory or targeted agents could be clinically relevant, although this requires prospective validation [60].

### Strengths, Limitations, and Future Directions

This study integrates systemic inflammatory indices, molecular alterations, and PD-L1 expression into a unified predictive model, offering a practical and accessible tool for estimating immunotherapy response in advanced NSCLC. The cohort was relatively large and drawn from a single tertiary oncology center with standardized diagnostic and therapeutic protocols, minimizing technical variability. Data were collected from real-world clinical practice, ensuring that findings reflect the biological and clinical heterogeneity encountered in daily oncology settings rather than the controlled context of clinical trials. Statistical analyses were methodologically robust, employing multivariate regression and ROC modeling to ensure internal consistency and reproducibility.

The main limitations arise from the retrospective and single-center design, which may restrict the generalizability of results. Molecular profiling was limited to the most common driver mutations, EGFR, KRAS, ALK, and TP53, without inclusion of co-mutations or extended genomic features such as STK11, KEAP1, or MET. Tumor mutational burden, microsatellite instability, and immune gene expression profiles were not assessed, precluding deeper molecular characterization. Inflammatory markers were evaluated at a single baseline time point, without longitudinal monitoring during therapy, which may underestimate their dynamic predictive value. Imaging-based assessment followed institutional standards and may not have fully captured atypical immunotherapy responses such as pseudo-progression. Potential selection bias inherent to retrospective data and the lack of an external validation cohort also represent relevant constraints.

Future research should validate these findings prospectively across multiple centers and populations. Longitudinal tracking of inflammatory indices during treatment could clarify how systemic inflammation evolves in relation to immune activation or exhaustion. Integration of next generation sequencing panels, circulating tumor DNA, and transcriptomic immune signatures could refine the biological precision of this model. Combining blood-derived biomarkers with radiomic, morphologic, or digital pathology features may further enhance predictive accuracy. Additionally, interventional trials targeting systemic inflammation or tumor-driven immune suppression could explore whether modulation of unfavorable inflammatory–molecular profiles improve clinical outcomes in immunotherapy-treated NSCLC.

## 5. Conclusions

The results of this study demonstrate that combining systemic inflammatory indices with molecular and immunohistochemical markers significantly improves the prediction of immunotherapy outcomes in advanced NSCLC. Patients with low NLR and high PD-L1 expression, lacking oncogenic driver mutations, achieved the highest response rates and longest survival, defining an immune-active phenotype with optimal checkpoint inhibitor sensitivity. In contrast, those with high NLR, low PD-L1 levels, and EGFR or ALK alterations exhibited markedly reduced response and early progression, confirming the immune-resistant profile of these subgroups. Intermediate clusters, characterized by KRAS or TP53 mutations and moderate inflammation, showed partial benefit, suggesting a transitional immunologic state influenced by both genomic and host-derived factors. Multivariate analysis identified NLR ≥ 5, PD-L1 < 1%, and EGFR mutation as independent predictors of poor progression-free survival. The integrated inflammatory–molecular model achieved superior discriminative accuracy (AUC = 0.82) compared with any individual biomarker, underscoring its potential clinical utility for refined patient stratification and treatment selection in real-world settings.

## Figures and Tables

**Table 1:** Inclusion and exclusion criteria.

Category	Inclusion Criteria	Exclusion Criteria
Diagnostic & Stage	Age ≥ 18 years Histologically confirmed NSCLC (adenocarcinoma, squamous, large cell) [17] Stage IIIB–IV disease (TNM 8th edition) [18] at the start of immunotherapy Measurable disease per RECIST 1.1 [19]	Small cell lung cancer Mixed histology with predominant SCLC component Non-epithelial tumors (NEC, sarcomas) Concurrent active malignancy requiring systemic therapy
Treatment	At least one cycle of an approved immune checkpoint inhibitor (pembrolizumab, nivolumab, atezolizumab, durvalumab, etc.) Immunotherapy given as monotherapy or combined with platinum-based chemotherapy Treatment administered at OncoHelp Timișoara (2022–2024)	Immunotherapy used exclusively in adjuvant or neoadjuvant settings Prior immunotherapy for another cancer before NSCLC diagnosis Enrollment in clinical trials with investigational combinations affecting systemic inflammation Major surgery or extensive radiotherapy within the past 4 weeks with persistent inflammation
Baseline Laboratory Data	Complete blood count (N, L, M, Plt, Eo) available between −7 and 0 days before first immunotherapy dose No acute events expected to significantly alter CBC	Documented acute infection (COVID-19, pneumonia, sepsis) within 14 days G-CSF administration in the past 14–21 days Blood transfusion within 14 days Chronic hematologic disorders (MDS, MPN, leukemia) History of splenectomy or hypersplenism
Autoimmune/Inflammatory Conditions	No active autoimmune disease requiring systemic immunosuppression No chronic immunosuppressive therapy	Autoimmune conditions requiring ≥10 mg prednisone/day Chronic immunosuppressive medication (MTX, AZA, MMF, TNF-α inhibitors, etc.) Transplant recipients on maintenance immunosuppression Uncontrolled chronic infections (HIV with severe immunosuppression, active HBV/HCV)
Molecular/IHC Data	PD-L1 TPS available (<1%, 1–49%, ≥50%) At least one molecular marker available (EGFR, KRAS, ALK, TP53)	Missing PD-L1 status Lack of all required molecular markers
Clinical Data & Follow-up	ECOG Performance Status documented Smoking status, comorbidities and demographics recorded Imaging available for response assessment at 9–12 weeks Adequate follow-up for PFS/OS (≥3 months unless early death)	Missing imaging required for response evaluation Lost to follow-up before first assessment Major inconsistencies in clinical documentation
Data Quality	Patients treated according to standard (non-experimental) protocols Medical record sufficiently complete for key study variables	Missing baseline CBC, PD-L1, molecular markers or imaging data Major non-adherence to therapy or follow-up

**Table 2:** Baseline demographic, clinical, and molecular characteristics.

Characteristic	Value
Age, years (mean ± SD)	65.1 ± 9.5
Sex, *n* (%)	Male: 210 (70.5%) Female: 88 (29.5%)
Smoking status, *n* (%)	Current/former: 214 (71.8%) Never: 84 (28.2%)
ECOG performance status 0–1, *n* (%)	189 (63.4%)
Histology, *n* (%)	Adenocarcinoma: 163 (54.7%) Squamous: 93 (31.2%) Large cell: 42 (14.1%)
Primary tumor location	Peripheral: 182 (61.1%) Central: 116 (38.9%)
PD-L1 expression, *n* (%)	<1%: 79 (26.5%) 1–49%: 111 (37.2%) ≥50%: 108 (36.3%)
EGFR mutation	14 (4.7%)
KRAS mutation	8 (2.7%)
ALK rearrangement	3 (1.0%)
TP53 alteration	39 (13.1%)
Comorbidities (≥1 major)	168 (56.4%)

Abbreviations: SD, standard deviation; *n*, number; ECOG, Eastern Cooperative Oncology Group; PD-L1, programmed death-ligand 1; EGFR, epidermal growth factor receptor; KRAS, Kirsten rat sarcoma virus; ALK, anaplastic lymphoma kinase; TP53, tumor protein p53.

**Table 3:** Baseline systemic inflammatory markers according to PD-L1 expression.

Inflammatory Marker	PD-L1 < 1% (*n* = 79)	PD-L1 1–49% (*n* = 111)	PD-L1 ≥ 50% (*n* = 108)	*p*-Value
NLR, IQR	6.0 (3.8–8.5)	5.0 (3.2–6.9)	3.8 (2.5–5.8)	0.018
PLR, IQR	255 (206–310)	243 (191–285)	218 (168–267)	0.032
LMR, IQR	2.0 (1.6–2.5)	2.3 (1.7–2.8)	2.8 (2.1–3.4)	0.011
SII, IQR	1180 (860–1650)	1020 (750–1340)	880 (670–1150)	0.024

Abbreviations: PD-L1, programmed death-ligand 1; NLR, neutrophil-to-lymphocyte ratio; PLR, platelet-to-lymphocyte ratio; LMR, lymphocyte-to-monocyte ratio; SII, systemic immune–inflammation index; IQR, interquartile range; *n*, number.

**Table 4:** Composition and defining features of inflammatory–molecular clusters.

Cluster	PD-L1 Profile	Dominant Molecular Alterations	Median NLR	Median LMR	Patients (*n*, %)
A	≥50%	Wild type	2.4	3.0	78 (26.2%)
B	1–49%	KRAS, TP53	6.0	2.1	71 (23.8%)
C	<1%	EGFR, ALK	6.8	1.8	59 (19.8%)
D	Mixed	None detected	4.2	2.6	90 (30.2%)

Abbreviations: PD-L1, programmed death-ligand 1; NLR, neutrophil-to-lymphocyte ratio; LMR, lymphocyte-to-monocyte ratio; EGFR, epidermal growth factor receptor; KRAS, Kirsten rat sarcoma virus; ALK, anaplastic lymphoma kinase; TP53, tumor protein p53; *n*, number.

**Table 5:** Response to immunotherapy by inflammatory–molecular cluster.

Cluster	(ORR, %)	(DCR, %)	Median PFS (Months)	Median OS (Months)
A	41.0	77.0	13.0	22.5
B	25.3	55.6	8.4	15.9
C	7.0	26.5	4.3	9.2
D	26.7	50.0	9.1	14.8
*p*-value	<0.001	<0.001	<0.001	0.003

Abbreviations: ORR, objective response rate; DCR, disease control rate; PFS, progression-free survival; OS, overall survival.

**Table 6:** Multivariate Cox proportional-hazards model for progression-free survival.

Variable	Hazard Ratio (HR)	95% Confidence Interval (CI)	*p*-Value
NLR ≥ 5	2.12	1.46–3.07	<0.001
PD-L1 < 1%	1.91	1.26–2.90	0.002
EGFR mutation	2.36	1.28–4.36	0.006
KRAS mutation	1.59	0.89–2.83	0.108
TP53 alteration	1.22	0.78–1.90	0.373
ECOG ≥ 2	1.64	1.05–2.57	0.028

Abbreviations: HR, hazard ratio; CI, confidence interval; NLR, neutrophil-to-lymphocyte ratio; PD-L1, programmed death-ligand 1; EGFR, epidermal growth factor receptor; KRAS, Kirsten rat sarcoma virus; TP53, tumor protein p53; ECOG, Eastern Cooperative Oncology Group.

## Data Availability

The data presented in this study are available upon request from the corresponding author. The data are not publicly available due to hospital policy.
